# Influence of body mass index on recurrence of atrial fibrillation after electrical cardioversion

**DOI:** 10.1371/journal.pone.0291938

**Published:** 2023-09-22

**Authors:** Carmen Ligero, Victor Bazan, José M. Guerra, Moisés Rodríguez-Mañero, Xavier Viñolas, Josep M. Alegret

**Affiliations:** 1 Hospital Universitari de Sant Joan, Universitat Rovira i Virgili, Reus, Spain; 2 Hospital Universitari Germans Trias i Pujol, Universitat Autònoma de Barcelona, Badalona, Spain; 3 Hospital de la Santa Creu i Sant Pau, Universitat Autònoma de Barcelona, Barcelona, Spain; 4 Hospital Clínico Universitario de Santiago, Universidad de Santiago, Santiago de Compostela, Spain; University of Minnesota, UNITED STATES

## Abstract

**Background:**

Several studies have shown an independent relationship between body mass index (BMI) and the incidence of atrial fibrillation (AF). However, little is known about the influence of BMI on AF recurrence after electrical cardioversion (ECV).

**Methods:**

We selected 1121 patients who reverted to sinus rhythm after scheduled ECV and were included in three prospective Spanish registries of ECV in persistent AF. The patients were classified according to baseline BMI into three categories (normal weight, overweight, obesity). We assessed the influence of BMI on the rate of AF recurrence at 3 months.

**Results:**

We identified 538 patients (48%) who had AF recurrence in the first 3 months after successful ECV. The patients who suffered AF recurrence had a higher BMI than those who remained in sinus rhythm (29.66±4.57 vs. 28.87±4.64 Kg/m2, respectively; p = 0.004). We observed a higher incidence of AF recurrence in the overweight and obese patients (BMI ≥25 kg/m^2^) than in those classified as normal weight (50.5% vs. 35.6%, respectively; p<0,001). BMI≥25 Kg/m^2^ was shown to be independently related to of AF recurrence in the multivariate analysis (OR = 1.75, 95% confidence interval = 1.20–2.58; p = 0.004).

**Conclusions:**

Increased BMI is independently related to AF recurrence after ECV. BMI should also be taken into account when making decisions about the indication for ECV in persistent AF.

## Introduction

AF (atrial fibrillation) is the most common sustained arrhythmia in clinical practice and is responsible for high morbidity and mortality. The increasing prevalence of AF worldwide, both in developed and developing countries, due to ageing is a matter of concern [1]. Therefore, a better understanding of the modifiable risk factors for this arrhythmia is necessary to curb AF incidence and the economic burden it entails [2].

Overweight and obesity are major risk factors for noncommunicable diseases such as cardiovascular diseases, which were the leading causes of death in 2019, as reported by the World Health Organization (WHO) [3, 4]. AF has a strong epidemiological association with obesity and overweight. The relationship between BMI and new-onset AF is well known, and the increased incidence of AF in obese and overweight patients has been widely reported [5–7].

Recent studies suggest a prognostic benefit of the rhythm control strategy in front of the rate control strategy [8]. ECV (electrical cardioversion) is a frequently used treatment in persistent AF within the rhythm control strategy, which also includes ablation and anti-arrhythmic drugs. However, the incidence of AF recurrence after ECV is very high. For this reason, proper selection of ECV candidates is crucial, taking into account the factors related to the risk of recurrence. In this sense, little is known about the relationship between BMI and AF recurrence after ECV. Knowing the influence of BMI on recurrence would be useful in improving the selection of ECV candidates.

In this study, we aimed to evaluate the impact of BMI on AF recurrence in a wide group of patients with persistent AF who were treated with ECV.

## Methods

### Study population

Data were extracted from three Spanish registries designed to monitor the clinical practice of ECV in persistent AF (REVERSE, REVERCAT, CARDIOVERSE) [9–11]. These registries prospectively and consecutively included patients referred for elective ECV at 99 Spanish hospitals. The requirements for inclusion were consistent between the three registries and required patients to be >18 years old, to have persistent AF, defined as an episode of arrhythmia lasting 7 or more days and no precipitating conditions such as hyperthyroidism, fever, recent thoracic surgery and pericarditis. The data recorded included the clinical characteristics, treatment details, echocardiography results, and ECV procedure variables.

Out of a total of 3263 candidates for ECV included in all 3 surveys, we selected those patients without structural heart disease who had a successful ECV and had a planned follow-up at 3 months. Successful ECV was considered when the patient was discharged after the procedure in sinus rhythm (SR). AF recurrence was defined as reappearance of arrhythmia at any time after discharge from ECV up to the time of the 3-month follow-up visit. ECG evidence was required for this diagnosis.

**Fig 1:**
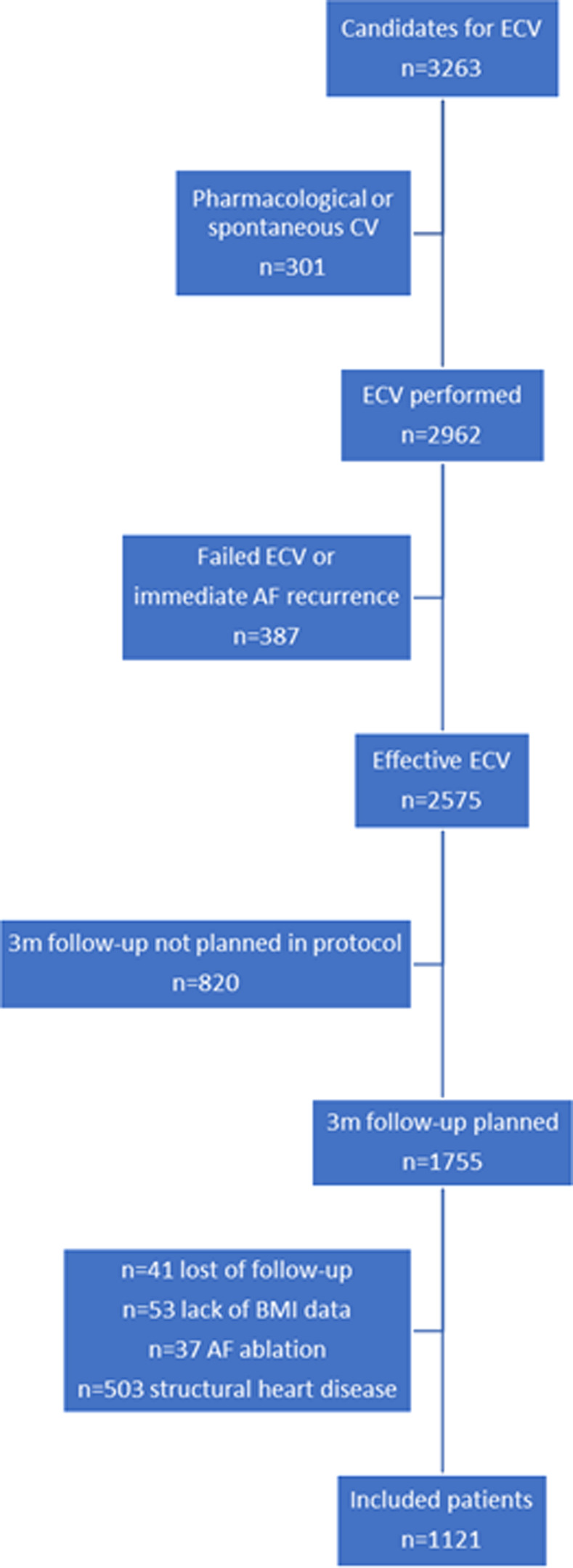
Consort flow chart representing selection of patients included.

Periprocedural antiarrhythmic drugs could be prescribed at the discretion of the physician, but those patients receiving antiarrhythmic drugs who reverted to SR prior to ECV were excluded from this analysis. Patients who underwent AF ablation and those with incomplete BMI data or follow-up were excluded. Consort flow chart about selection of patients included is presented in Fig 1.

Structural heart disease was considered in the presence of any of the following anomalies: moderate or severe valvular heart disease; mitral stenosis of any grade; previous myocardial infarction; systolic dysfunction (EF <50%); or any cardiomyopathy. Because of the date of the studies, the CHA2DS2VASC score was recorded only in the CARDIOVERSE study [11].

According to the WHO criteria, normal weight was defined when BMI was in the range between 18.5 and 24.9 kg/m2, overweight was defined when BMI was in the range of 25 to 29.9 kg/m2, and obesity when a BMI was greater than or equal to 30 kg/m2 [3]. We did not consider the subgroups of obesity (class 1, 2 and 3) by significantly reducing the number of patients per group.

### Statistical analysis

Normally distributed continuous quantitative variables are presented as the mean ± standard deviation (SD). Categorical variables are expressed as frequencies and percentages.

Comparison between the group of patients who remained in SR versus those who presented AF recurrence during follow-up was studied using a Chi-square test.

Correlations between BMI and AF recurrence in the subgroups were assessed using the Kendall rank correlation coefficient. Univariable and multivariable regression analyses were performed to assess predictors of AF recurrence at baseline (logistic regression) among the anthropometric parameters, specifically BMI. Receiver operating characteristic analysis and the discriminative ability by the area under curve (AUC) for predicting AF recurrence were calculated for BMI.

Likewise, a stratified analysis was executed by the different BMI categories based on the WHO definition [3] to compare overweight and obese patients (BMI 25–29.9 and BMI≥30 kg/m2, respectively) with those classified as normal BMI (18.5–24.9 kg/m2).

Statistical significance was set at a p value < 0,05.

Statistical calculations were made using IBM SPSS Statistics for Windows, Version 29.0.

The studies were conducted according to the principles of the Declaration of Helsinki and were approved by the Institutional Review Board and Ethics Committee of the Hospital Universitari de Sant Joan (main board) and posteriorly by the rest of the participant centres. The participants provided informed written consent prior to participating in these studies.

## Results

### Patients’ characteristics

A total of 1121 patients were included. The clinical and echocardiographic characteristics of the study population are shown in Table 1. The mean age was 63.5 ± 10.6 years. Most of the patients were male (69.3%) and had good functional status at baseline (68.8% NYHA functional class I). More than half suffered from hypertension (54.4%), and the prevalence of long-term AF was low (15%). Regarding echocardiographic characteristics, there was a low proportion of patients with significant left atrial enlargement (12.3%). The vast majority of patients (80.3%) were treated with antiarrhythmic drugs, and most of the patients were overweight (45.3%) or obese (38%).

**Table 1:** Baseline clinical and echocardiographic characteristics of the study population.

Characteristics		n (%) or mean (SD)
Age; years		63.51 (10.6)
Sex (male)		777 (69.3)
Height; cm		168.02 (9.19)
Weight; Kg		82.5 (14.25)
Body surface; m^2^		1.92 (0.19)
Body mass index; Kg/m^2^		29.22 (4.58)
Normal weight (18.5–24.9 Kg/m2)		187 (16.7)
Overweight (25–29.9 Kg/m2)		508 (45.3)
Obesity (≥ 30 Kg/m2)		426 (38)
Hypertension		610 (54.4)
Diabetes mellitus		180 (16.1)
COPD		84 (7.5)
Previous embolism		64 (5.7)
Previous ECV		198 (17.7)
AF duration > 1 year		168 (15,0)
NYHA functional class	I	772 (68.8)
	≥ II	349 (31.2)
LA AP diameter (mm)		43,37 (5.5)
LA AP diameter ≥ 50 mm		138 (12.3)
LVEF (%)		61.55 (8.15)
Antiarrhythmic drugs post-ECV		900 (80.3)
Amiodarone		616 (55)
Ic class antiarrythmic drugs		278 (24.8)
Other antiarrythmic drugs		6 (0.5)

AF = atrial fibrillation; AP = anteroposterior; COPD = chronic obstructive pulmonary disease; ECV = electrical cardioversion; LA = left atrium; LVEF = left ventricle ejection fraction; NYHA = New York Heart Association

### BMI and AF recurrence

A total of 538 patients (48%) of the 1121 included in our analysis were diagnosed with AF recurrence within 3 months after successful ECV. We observed a higher BMI among those patients who suffered an AF recurrence compared to those who remained in SR (29.66±4.57 kg/m2 vs. 28.87±4.64 kg/m^2^, respectively; p = 0.004) (Table 2).

**Table 2:** Patient characteristics by rhythm at 3 months post-ECV.

Characteristics n (%) or mean (SD)	AF recurrence	Sinus rhythm	p
Age; years	63.53 (9.59)	63.44 (11.59)	0.890
Sex (male)	369 (68.6)	408 (70.0)	0.582
Height; cm	167.94 (9,14)	167.98 (9,17)	0.943
Weight; Kg	83.61 (13.75)	81.54 (14.87)	0.016
Body surface area; m^2^	1.930 (0.18)	1.909 (0.20)	0.067
Body mass index; Kg/m^2^	29.66 (4.57)	28.87 (4.64)	0.004
Body mass index ≥25 Kg/m^2^	472 (87.7)	462 (79.2)	<0.001
LA AP diameter; mm	43.58 (5.14)	43.10 (5.79)	0.175
LVEF (%)	61.56 (7.94)	61.53 (8.17)	0.963
Hypertension	296 (55)	314 (53.9)	0.744
Diabetes mellitus	91 (16.9)	89 (15.3)	0.460
COPD	40 (7.4)	44 (7.6)	0.896
AF duration >1 year	91 (16.9)	77 (13.2)	0.085
Previous ECV	92 (17.1)	106 (18.2)	0.647
Previous embolism	32 (5.9)	32 (5.5)	0.751
Antiarrhythmic drugs	402 (74.7)	498 (85.4)	<0.001
Amiodarone	268 (49.8)	348 (59.7)	0.001
Ic	128 (23.7)	150 (25.7)	0.453

AF = atrial fibrillation; AP = anteroposterior; COPD = chronic obstructive pulmonary disease; ECV = electrical cardioversion; LA = left atrium; LVEF = left ventricle ejection fraction

**Fig 2:**
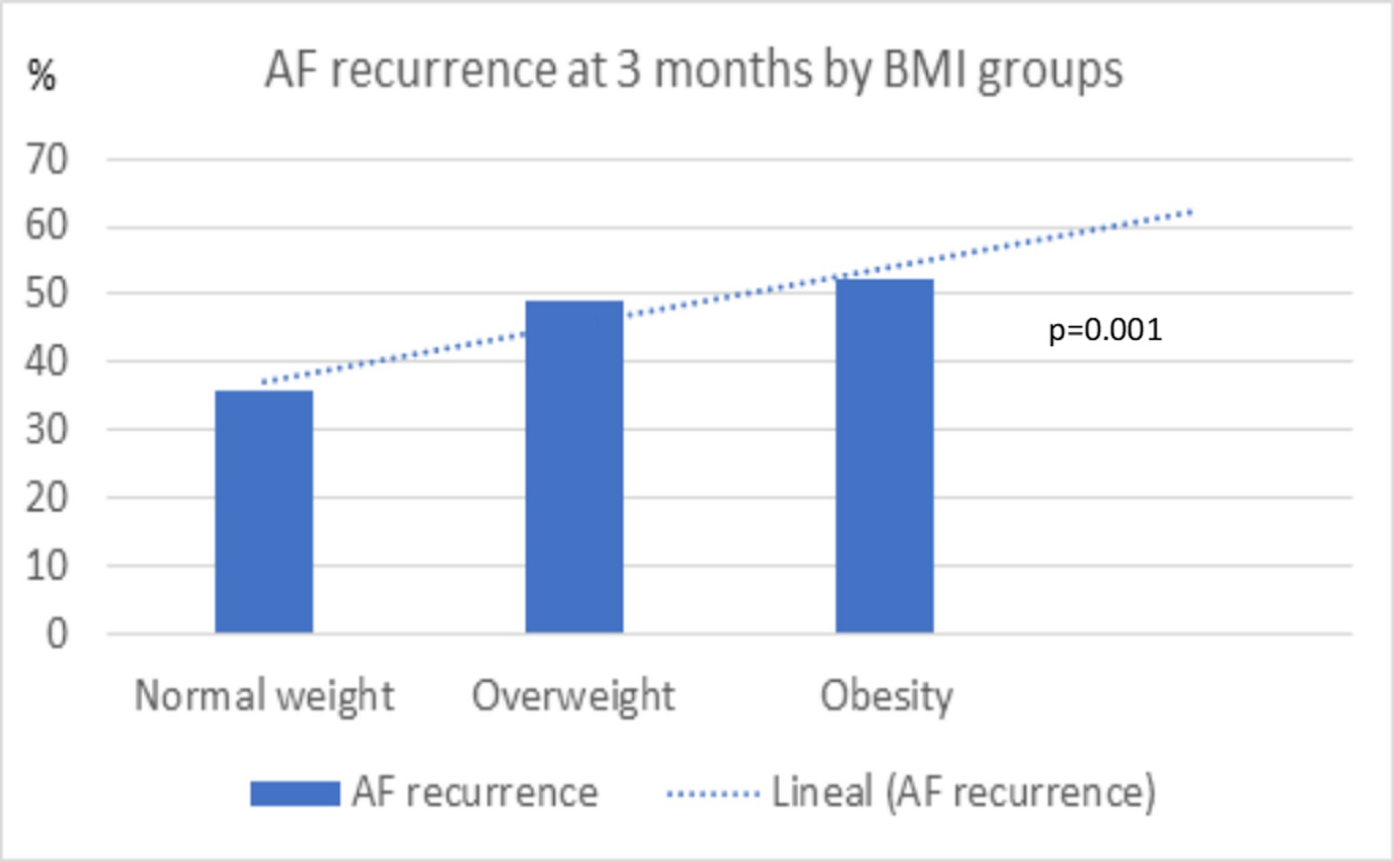
Incidence of atrial fibrillation recurrence and lineal correlation in the 3 groups: Normal weight, overweight, obesity.

The patients with an elevated BMI showed more AF recurrence at follow-up. Among the patients with BMI in the range of overweight and obesity, we observed a higher incidence of AF recurrence with respect to the patients with normal weight (50.5% vs. 35.6%, respectively; p<0,001). In addition, we observed progressively higher frequencies of AF recurrence in the overweight and obese categories compared to the lower category of normal weight (normal weight 35.6%, overweight 49.1%, obesity 52.1%; p = 0.001; normal weight vs. overweight p = 0.001; normal weight vs. obesity p<0.001; Fig 2). We further assessed the discriminating ability of BMI to predict AF recurrence, showing an AUC of 0.57 (95% CI, 0.54–0.60, p = 0.001). Different cutoff points with similar precision were observed between 24.8 Kg/m^2^ and 29 Kg/m^2^. We chose 25 kg/m^2^ as the cut-off point to consider BMI as a qualitative variable since we considered it to be the one with the greatest clinical value, when defining increased BMI.

**Fig 3:**
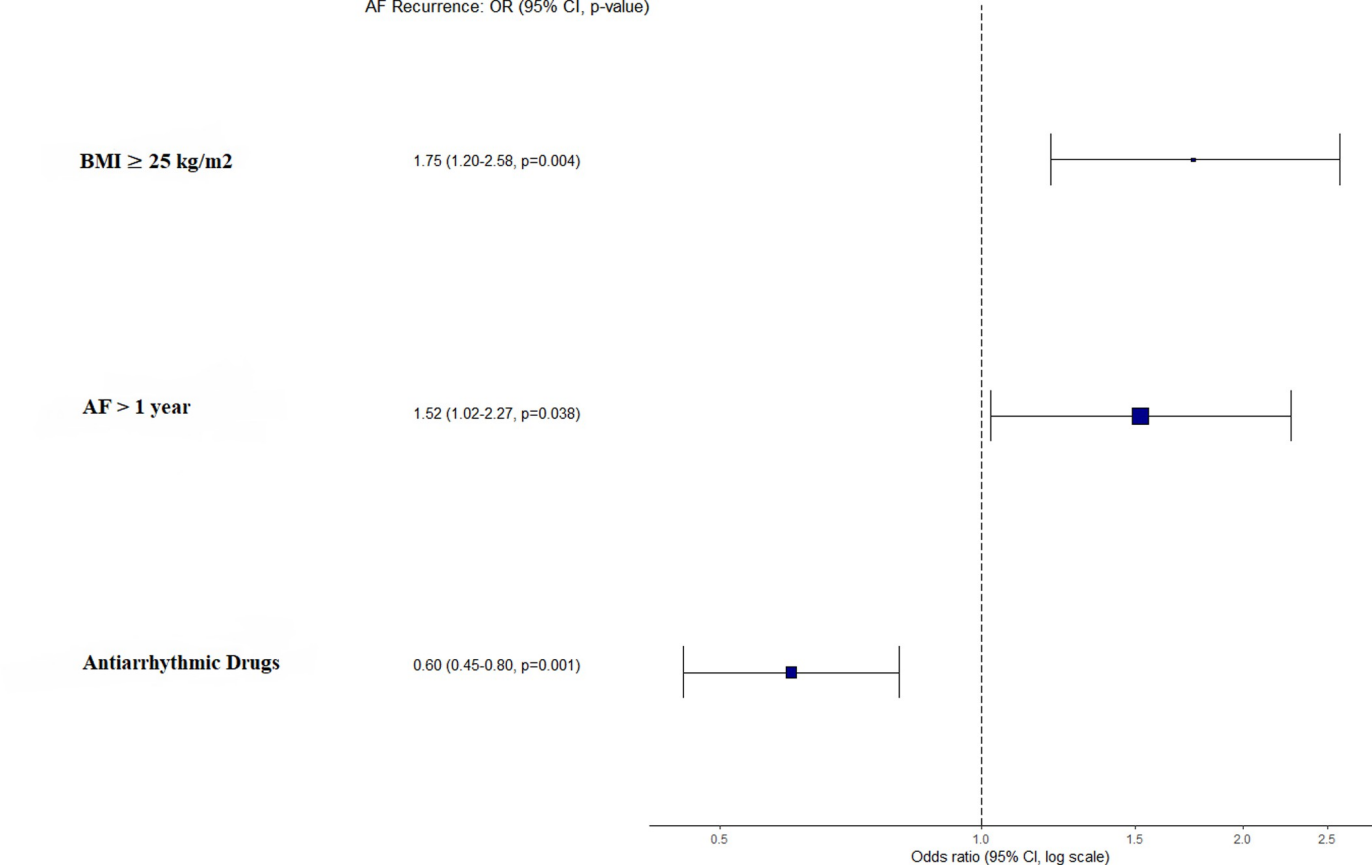
Representation of odds ratio and 95% confidence interval of those variables included in the logistic regression.

We performed a multivariate analysis (logistic regression model) to assess whether BMI ≥25 kg/m^2^ was independently related to AF recurrence. BMI ≥25 kg/m^2^ emerged as an independent variable related to AF recurrence (OR = 1.75, 95% confidence interval = 1.20–2.58; p = 0.004), as well as other known factors related to AF recurrence (use of antiarrhythmic drugs, AF duration > 1 year) (Fig 3).

## Discussion

In this study, we observed that in patients with persistent AF, BMI was directly related to a higher rate of AF recurrence after ECV. An elevated BMI increased the risk of AF recurrence after ECV, with a progressively increased risk in overweight and obese patients with respect to normal weight patients. A BMI ≥ 25 kg/m2 was independently related to AF recurrence after adjusting for other factors. Thus, an increased BMI should be taken into account in the decision-making of ECV in patients with persistent AF.

Different epidemiological studies have previously described the relationship between BMI and the incidence of new-onset AF. Overweight and obesity have been described to increase the risk for new-onset AF by 12.3% and 32.7%, respectively. Some structural, functional, metabolic and neurohumoural changes induced by obesity affecting the heart have been proposed as potential causes. Obesity is related to the enlargement of the cardiac cavities, including atrial remodelling [12, 13], a fact related to both the development and persistence of AF [14–17]. Multitude neurohumoural and metabolic alterations observed in obesity could also contribute to changes in cardiac structure and function. These alterations include increased insulin resistance, activation of the renin-angiotensin-aldosterone system and arterial hypertension [12]. The renin-angiotensin-aldosterone system is recognized to play a key role in the induction of cardiac inflammation and fibrosis, the basis of atrial and ventricular electrical and structural remodelling. These changes give rise to alterations in ions and cell junctions that lead to the development of AF [18, 19]. On the other hand, the volume of epicardial adipose tissue (EAT) has been related to the incidence of AF [20]. The association between BMI and visceral fat, specifically epicardial fat, has already been described [21]. Given its proximity to the heart, the influence of epicardial fat on arrhythmogenesis has aroused great interest in recent years. EAT has endocrine activity that could contribute to structural remodelling of the heart and specifically of the atrial myocardium. Atrial fibrosis can be induced by the paracrine action of adipocytokines secreted by EAT, potentiated by fatty infiltration of myocardial tissue and inflammation leading to fibrotic remodelling of adipose tissue in the atrial epicardium [22, 23]. Obesity-associated sleep disorders such as sleep apnoea also contribute to autonomic dysfunction due to hypoxia and acidosis. Mechanisms of arrhythmogenesis include abnormal automaticity, triggered automaticity, and re-entry mechanisms [24].

Nevertheless, it should be noted that the observed associations could not be well determined due to the limitations of conventional statistical methods, namely, potential confounders, such as the presence of structural heart disease, left atrial size or hypertension. In our study, after excluding patients with structural heart disease and adjusting for other related variables, overweight and obesity remained variables related to a higher risk of AF recurrence.

Previously, BMI has been described as a predictive factor for AF recurrence after ablation, mainly in the range of obesity [25, 26]. In our study, for patients treated with ECV, the influence of BMI on the increased risk of AF recurrence was evident since the range of overweight. From our study and from those previous studies on postablation recurrences, it is clear that it is important that patients who are chosen for a rhythm control strategy follow strict control of cardiovascular risk factors, specifically adequate control of metabolic factors and weight. All of this would contribute to increasing the chances of success of the rhythm control strategy. Likewise, given the high rate of post-ECV recurrences, overweight and obesity must be taken into account when making decisions about whether the best strategy to choose in a given patient is the rhythm control strategy or the heart rate control strategy.

Our study has several limitations. First, it has the limitations of a post hoc analysis. With respect to AF diagnosis, AF recurrence was diagnosed on the basis of 12-lead surface electrocardiograms. Silent self-limited AF episodes during the follow-up period could have been detected with prolonged ECG monitoring devices, but the main clinical interest was to identify the recurrence of persistent AF. We are aware that assessment of overweight or obesity through BMI is a limited approach since it does not measure the amount of body fat or its distribution. Obstructive sleep apnoea syndrome (OSAS) has been associated with an increased risk of AF recurrence after catheter ablation [27]. OSAS was only recorded in one of the registries that are part of our study [11]. It involved only a small number of patients (36 patients), so the effect of OSAS on AF recurrence could not be analyzed. On the other hand, underweight has also been reported to significantly increase the risk of AF recurrence post ablation [28]. The number of patients with underweight candidates to be included in our study was also very low (16 patients) to allow a subgroup analysis, and by this reason were excluded from the study.

However, we believe that the strengths of our study are its size and its observational nature, providing insight into the real world.

## Conclusions

In our study, an increase in BMI in the range of obesity and overweight was associated with higher AF recurrence in patients undergoing ECV. Based on our results, we postulate that high BMI should be taken into account as a risk factor for recurrence of AF. Consequently, a weight control strategy should be considered in the management of patients with persistent AF who are indicated for elective ECV.
